# Structural and functional analyses of the optic nerve and lateral geniculate nucleus in glaucoma

**DOI:** 10.1371/journal.pone.0194038

**Published:** 2018-03-23

**Authors:** Rafael Lacerda Furlanetto, Sergio Henrique Teixeira, Carolina Pelegrini Barbosa Gracitelli, Claudio Luiz Lottenberg, Fabiano Emori, Michel Michelan, Edson Amaro, Augusto Paranhos

**Affiliations:** 1 Department of Ophthalmology, Federal University of Sao Paulo, Sao Paulo, SP, Brazil; 2 Hospital Israelita Albert Einstein, Sao Paulo, SP, Brazil; Bascom Palmer Eye Institute, UNITED STATES

## Abstract

**Purpose:**

To analyze the correlation between structural characteristics of intraorbital optic nerve (ION) and lateral geniculate nucleus (LGN) measured by 3-Tesla magnetic resonance imaging (3T MRI), and the severity of glaucomatous damage.

**Methods:**

In this cross-sectional study, 41 glaucoma patients and 12 age- and sex-matched controls underwent standard automated perimetry (SAP) and frequency doubling technology (FDT) as functional evaluation; optic disc stereophotograph, spectral-domain optical coherence tomography (OCT) and confocal scanning laser tomography as ocular structural evaluation; and 3T MRI. Structure-structure and structure-function correlation were performed using bootstrap resampling method for clustered data.

**Results:**

The ION mean diameter and cross-sectional area were different between glaucoma and control groups at 5mm and 10mm (all, p≤0.011) from the globe, but not at 15mm (both, p≥0.067). LGN height was significantly lower in glaucoma group (p = 0.005). OCT rim area and functional parameters (SAP and FDT) correlated significantly with all ION segments, showing stronger correlations at 10 and 15 mm. ION parameters at 10 and 15 mm presented mild-to-moderate correlation with OCT peripapillary nerve fiber layer thickness, and ION at 15mm had mild association with the neuroretinal rim area on stereophotographs. Although LGN height was significantly smaller in glaucoma group (p = 0.005), LGN parameters were not associated with any ocular structural or functional parameter.

**Conclusion:**

Assessment of central and peripheral nervous systems using 3T MRI confirmed that glaucoma patients had smaller ION dimensions and LGN height compared to the control group. In general, ION dimensions presented mild to moderate correlations with functional and ocular structural parameters. Although ION had significant correlations at any distance from the eye, the ION distal locations correlated better with OCT results and functional parameters. However, LGN parameters were not associated with functional or ocular structural parameters.

## Introduction

Glaucoma is a degenerative optic neuropathy characterized by progressive loss of retinal ganglion cells and their axons, resulting in characteristic changes at the optic nerve head (ONH) and correspondent visual field loss.[1, 2] Previous research in experimental and human glaucoma demonstrated degeneration of structures from the anterior visual pathway, including the optic nerve and the lateral geniculate nucleus (LGN).[3–11]

In the anterior visual pathway, there are different types of retinal ganglion cells that comprise separate paths and are named according to their targets in the LGN. In general, the parvocellular pathway accounts for 80% of the ganglion cells within the optic nerve, whereas approximately other 10% comprise the magnocellular pathway.[12] Although glaucoma leads cells from both magnocellular and parvocellular visual pathways to atrophy, some investigators suggested a preferential damage of larger axons in the optic nerve in experimental glaucoma,[5, 6] and a more prominent cell loss was reported on magnocellular than on parvocellular layers of the LGN.[9, 10] However, such alterations at the LGN were not confirmed by other researchers,[13] therefore, whether one of those pathways is preferentially affected in living human glaucoma still remains controversial.

For glaucoma assessment *in vivo*, qualitative and quantitative structural analyses are mostly limited to parameters of intraocular structures, such as those from the ONH and the retinal nerve fiber layer (RNFL), whereas visual function depends on the integrity of the entire optic pathway. In this scenario, new methods to assess and monitor glaucomatous structural changes in the central nervous system (CNS) could be useful.

Magnetic resonance imaging (MRI) has been largely used as a sensitive and non-invasive imaging modality to assess intracranial structures. High-speed image acquisition and enhanced spatial resolution scans were incorporated in the modern 3-Tesla (3T) MRI devices and allowed accurate measurements of the intracranial structures.[14–16] Different studies using previous versions of MRI systems reported atrophy of the intraorbital optic nerve (ION) and the LGN in glaucoma,[17–19] and MRI measurements were consistent with histological analyses.[7, 20] Nevertheless, only few studies investigated the correlation between outcomes from ocular tests and 3T MRI structural findings of the anterior visual pathway in human glaucoma.[15, 16, 21, 22] Although some significant associations were reported, such correlation analyses involving the ION and/or the LGN remain unclear and need further clarification, given the differences among those studies regarding sample characteristics (including age of participants and stages of glaucomatous damage), MRI data acquisition protocols and statistical models applied. In addition, none of those studies considered the subtypes of visual pathway in their structure-function correlation analyses.

The purpose of our study was to scrutinize the existence and the strength of the associations between MRI-defined structural parameters of the ION and LGN, and structural and functional parameters conventionally used for glaucoma evaluation.

## Material and methods

This was a prospective, cross-sectional study approved by the institutional ethics committees of the Federal University of Sao Paulo and the Albert Einstein Israeli Hospital (Sao Paulo, Brazil). Informed consent was obtained from all participants and the study was performed in accordance with the tenets of the Declaration of Helsinki.

### Participants

We recruited 41 primary open-angle glaucoma (POAG) patients with a wide range of ONH and visual field damage, and 12 age- and sex-matched healthy volunteers from the Department of Ophthalmology of Federal University of Sao Paulo, Brazil. Mean age was 62.9±7.0 years in the glaucoma group and 63.2±5.7 years in the control group (p = 0.898). Table 1 summarizes demographic and clinical characteristics of the sample. Glaucoma was defined based upon the clinical determination of glaucomatous ONH damage (localized or diffuse neuroretinal rim thinning, rim notching, excavation, and/or RNFL defect) associated with typical, reproducible standard automated perimetry (SAP) defects.[1, 2] Glaucomatous defect on SAP was defined based upon a glaucoma hemifield test result outside normal limits and the presence of at least 3 contiguous test points within the same hemifield on the pattern deviation plot at P<1%, with at least 1 point at P<0.5%, on at least 2 consecutive tests, with reliability indices better than 15%.

**Table 1:** Clinical and demographic characteristics of the study population.

Variables	Glaucoma	Control	P Value
	(n = 41)	(n = 12)	
Age, mean (SD), years	62.9 (7.0)	63.2 (5.7)	0.898*
Gender, n (%)			0.492^†^
- Female	23 (56.1%)	6 (50%)	
- Male	18 (43.9%)	6 (50%)	
Ethnics, No. (%)			0.001^†^
- Caucasian	22 (53.7)	7 (58.3)	
- African descent	7 (17.1)	3 (25)	
- Mixed	11 (26.8)	2 (16.7)	
- Asian descent	1 (2.4)	0 (0)	
IOP, mean (SD), mmHg	15.2 (3.7)	14.6 (2.2)	0.483^‡^
Central Corneal Thickness, mean (SD), μm	523.5 (35.2)	528.6 (25.6)	0.444^‡^
Spherical equivalent, mean (SD), diopters	+0.7 (1.56)	+0.95 (1.27)	0.387^‡^
BCVA, mean (SD), logMAR	0.1862 (0.18)	0.0037 (0.02)	0.024^‡^
Optic Disc Stereophotograph			
- Vertical CDR, mean (SD)	0.8 (0.12)	0.44 (0.09)	<0.001^‡^
- Average CDR, mean (SD)	0.81 (0.1)	0.48 (0.09)	<0.001^‡^
- Rim Area, mean (SD), mm^2^	0.82 (0.43)	1.69 (0.24)	<0.001^‡^
Optical Coherence Tomography			
- RNFLT, mean (SD), μm	64.7 (10.6)	95.4 (6.9)	<0.001^‡^
- Vertical CDR, mean (SD)	0.81 (0.08)	0.52 (0.11)	<0.001^‡^
- Average CDR, mean (SD)	0.82 (0.07)	0.54 (0.12)	<0.001^‡^
- Rim Area, mean (SD), mm^2^	0.65 (0.24)	1.3 (0.15)	<0.001^‡^
CSLO			
- Cup/Disc Area Ratio, mean (SD)	0.62 (0.16)	0.24 (0.14)	<0.001^‡^
- Linear CDR, mean (SD)	0.78 (0.12)	0.47 (0.15)	<0.001^‡^
- Rim Area, mean (SD), mm^2^	0.88 (0.36)	1.5 (0.26)	<0.001^‡^
SAP MD, mean (SD), dB	-15.39 (10.02)	-0.38 (0.89)	<0.001^‡^
SAP VFI, mean (SD), %	56.6 (32.7)	99.3 (0.8)	<0.001^‡^
FDT MD, mean (SD), dB	-13.67 (7.79)	+1.74 (2.1)	<0.001^‡^

* *t* test.

† χ^2^ test.

‡ Generalized estimating equations.

SD = Standard deviation; IOP = intraocular pressure; BCVA = best corrected visual acuity; logMAR = logarithm of the minimum angle of resolution; CDR = cup-to-disc ratio; RNFLT = retinal nerve fiber layer thickness; CSLO = confocal scanning laser ophthalmoscopy; SAP = standard automated perimetry; MD = mean deviation; FDT = frequency doubling technology

All participants had a detailed medical history and underwent best-corrected visual acuity (BCVA) assessment, slit-lamp biomicroscopy, automated keratometry, axial length measurement by partial coherence laser interferometry, ultrasonic central corneal thickness, Goldmann applanation tonometry, gonioscopy, and stereoscopic optic disc examination. Inclusion criteria comprised an age range between 30 and 75 years. Ocular exclusion criteria for either eye of each participant included BCVA worse than 20/100, refractive error greater than ±5 diopters of sphere or 3 diopters of cylinder, optically significant cataract, gonioscopy showing occludable angle or peripheral anterior synechiae or excessive pigmentation or deposits of exfoliation material, history of inflammatory eye disease, prior ocular trauma, diabetic retinopathy, or other ocular or systemic diseases capable of causing visual field loss or optic nerve deterioration, including intracranial lesions or orbital diseases. Participants that presented insufficient cooperation during psychophysical tests or had claustrophobia, metallic implants, foreign bodies, tattoos and/or permanent makeup were also excluded. History of uneventful cataract surgery was allowed for all participants and, for glaucoma group, uncomplicated IOP-lowering surgical procedures were not prohibitive. Glaucoma patients had to present the disease in both eyes to be included in the study. Healthy volunteers were required to have, in both eyes, IOP <21 mmHg, clinically normal optic discs, SAP within normal limits, and no ocular or systemic abnormalities that could affect the optic nerve structure or visual function. Participants were investigated using the following techniques, and the interval between the first and the last exam (including 3T MRI) was less than 15 days.

### Functional testing

Visual function was evaluated using 2 different psychophysical tests. SAP was performed using 24–2 Swedish Interactive Threshold Algorithm (Humphrey Field Analyzer II, Carl Zeiss Meditec, Dublin, USA). To confirm an existing visual field defect, SAP was repeated within a 7-day period. Global indices, such as Mean Deviation (MD) and Visual Field Index (VFI), of the second reliable SAP test were selected for statistical analysis. The severity of glaucoma damage was classified as early, moderate or advanced in accordance with Hodapp-Parrish-Anderson criteria for SAP testing.[23] To improve the analysis and comprehension of the glaucoma group profile, patients were assigned into three subgroups according to the pattern of visual field defects in both eyes: (1) mid-stage glaucoma, composed by patients with early or moderate glaucoma in both eyes; (2) asymmetrical glaucoma, composed by patients in which one eye showed early glaucoma and the fellow eye had advanced glaucoma; and (3) advanced glaucoma, composed by patients whose one eye had advanced glaucoma and the fellow eye showed, at least, moderate glaucoma.

Frequency Doubling Technology (FDT) (Humphrey Matrix FDT Visual Field Instrument; Carl Zeiss Meditec, Dublin, USA) was performed using 24–2 threshold strategy with the Zippy Estimation by Sequential Testing (ZEST) algorithm. Details of the technique are described elsewhere.[24, 25] In brief, FDT is a perimetric test based on the frequency-doubling illusion and measures the contrast necessary to detect vertical grating targets that undergo counter-phase flicker.[24] Each target encompasses 5° of visual angle, has a spatial frequency of 0.5 cycles/degree and counter-phases in a temporal frequency of 18Hz. Similarly to SAP testing, FDT was performed twice for each patient within a 7-day period to confirm findings, and both FDT tests required reliability indices better than 20% to be included in the study. The results of the second reliable FDT test were selected for statistical analysis.

SAP and FDT outcomes (for instance, pointwise sensitivity values and MD) are usually provided in logarithmic scale [decibels (dB)]. In order to also investigate linear-to-linear relationships, we converted the pointwise sensitivity values, given in logarithmic scale (dB), to linear scale [for SAP, dB = 10 log10 (1/L), where L is light stimulus intensity as measured in Lamberts; and for FDT, dB = 20 log10 (1/C), where C is the reciprocal of the Michelson contrast], as recommended by previous investigators.[24, 26, 27] These values (in linear scale) were then averaged to obtain new parameters: the global mean sensitivity (named ‘Linear MS’), the temporal mean sensitivity, and the nasal mean sensitivity. To analyze specifically structure-function relationships involving LGN, two new functional parameters were then derived, once LGN receives afferents from the temporal retina (nasal hemifield) of the ipsilateral eye and from the nasal retina (temporal hemifield) of the contralateral eye: the ipsilateral mean sensitivity in linear scale (named ‘Ipsilateral Linear MS’), calculated by averaging the ipsilateral nasal mean sensitivity and the contralateral temporal mean sensitivity; and the contralateral mean sensitivity in linear scale (named ‘Contralateral Linear MS’), estimated from the average of the contralateral nasal mean sensitivity and the ipsilateral temporal mean sensitivity, both in linear scale. ‘Ipsilateral Linear MS’ and ‘Contralateral Linear MS’ were also converted back to logarithmic (dB) scale.

### Structural evaluation

Participants underwent non-simultaneous color optic disc stereophotograph imaging (FF450 plus IRu Retina Camera, Visupac software version 4.4, Carl Zeiss Meditec AG, Jena, Germany) after pupil dilation. The boundaries and the cup of the ONH were delineated by an experienced ophthalmologist (SHT) with a stereo-viewer, masked to participants’ clinical data, using the retinal camera built-in software. Automated image processing provided disc area, cup area, average cup-to-disc ratio (CDR), and CDR in different axes. Rim area was calculated as the difference between disc area and cup area. To avoid measurement bias associated with axial length-related ocular magnification, we applied the Littmann’s formula (*t* = *p* * *q* * *s*) as modified by Bennet.[28, 29] In brief, this formula is based on the assumption that the actual size of a fundus feature (*t*) is proportional to the magnification of the fundus imaging device (factor *p*), the optical dimensions of the eye (*q*) and the fundus feature measurement provided by the camera image software (*s*). The numerical factor *p* is instrument-dependent and, as proposed by Littmann, it is 1.37 for fundus cameras.[30] The ocular magnification factor related to the eye dimension (*q*) can be calculated as q = 0.01306 * (axial length– 1.82).[29] Once Littmann’s formula applies only to linear magnification, area measurements were corrected to *t*^2^ = *p*^2^ * *q*^2^ * *s*^2^, where *s*^2^ is the area measured with the fundus camera image (ie, disc area or rim area).[29]

Analysis of the ONH topography was performed using confocal scanning laser ophthalmoscopy (CSLO) [Heidelberg Retina Tomograph III (HRT 3); Heidelberg Engineering, GmbH, Dossenheim, Germany] in a dark room, without pupil dilation. To minimize measurement bias due to inadequate ocular magnification correction, spherical equivalent and keratometry measurements were properly set before CSLO image acquisition.[31] Three scans centered on the ONH were obtained for each eye and averaged by HRT 3 software (Heidelberg Eye Explorer version 1.5.1.0; Heidelberg Engineering, GmbH, Dossenheim, Germany) to create a single mean topography image. This image acquisition procedure was repeated three times for each eye, thus resulting in three different mean topography images. An experienced clinician (RLF) judged the one with the best image quality and overall quality score, and then outlined the optic disc margins. Once the contour line was drawn, the inbuilt CSLO software automatically generated ONH stereometric parameters including disc area, rim area, linear CDR, and cup-to-disc area ratio. Scans with Topography Standard Deviation index greater than 50 μm were excluded.

Spectral-domain optical coherence tomography (OCT) imaging was performed using Cirrus HD-OCT (version 5.0, Carl Zeiss Meditec, Inc., Dublin, CA) under pupil dilation using the Optic Disc Cube 200 x 200 protocol. In brief, this protocol scans a 200 X 200 X 1024-point parallelepiped (27,000 A-scans/sec), and the inbuilt software automatically calculates the peripapillary retinal nerve fiber layer thickness (RNFLT) and ONH parameters, including rim area, disc area, average CDR and vertical CDR. Only good-quality scans, defined as scans with a signal strength of ≥7, without RNFL discontinuity or misalignment, or involuntary saccade or blinking artifacts, and absence of RNFL algorithm segmentation failure, were used for analysis. Artificial tears were used before CSLO and OCT image acquisition for patient comfort.

### MRI data acquisition

**Fig 1:**
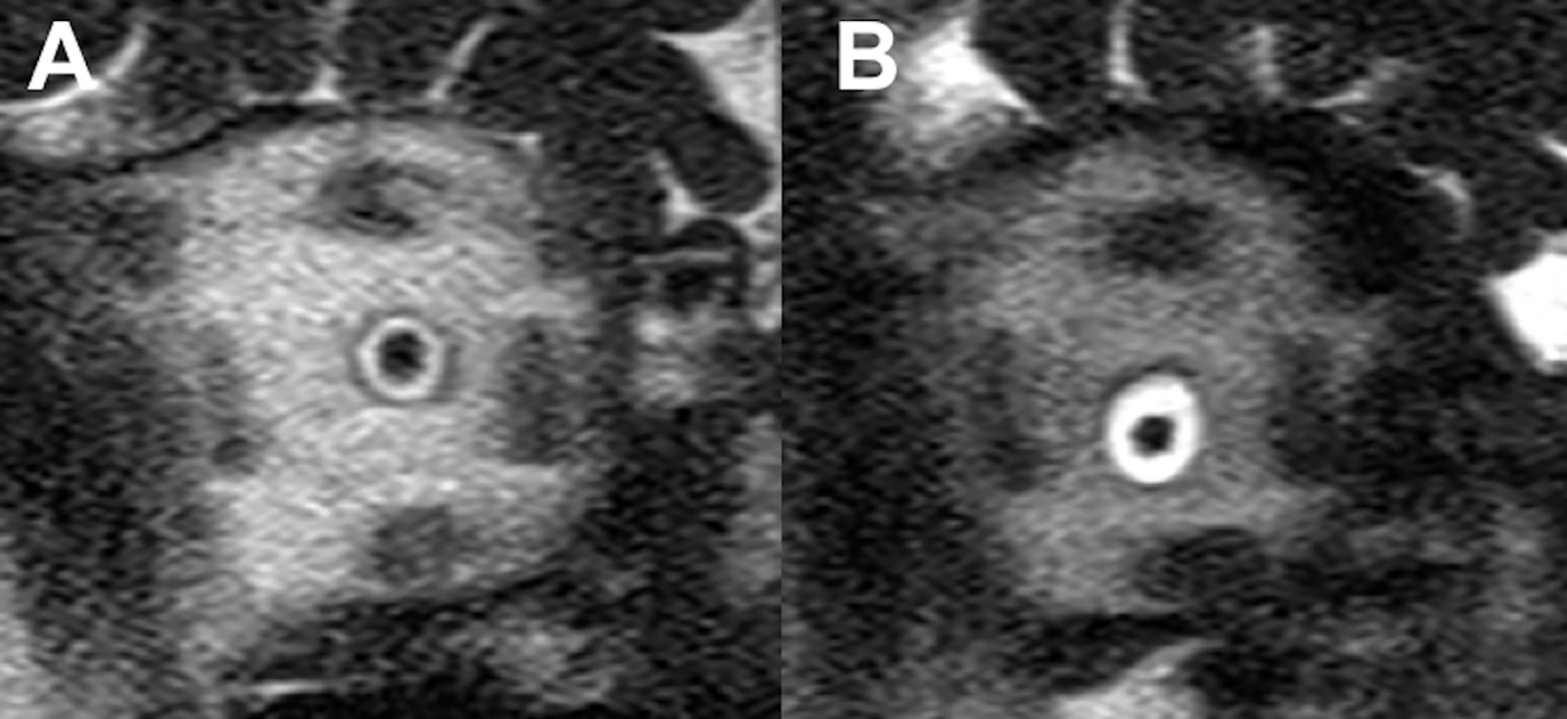
T2-weighted half-Fourier acquisition in single shot turbo spin-echo (HASTE) magnetic resonance imaging scans showing the cross-sectional area of the right intraorbital optic nerve (ION) 5 mm behind the eye. The cerebrospinal fluid can be identified as the hyperintense (bright) ring-shaped area in the center of the scan, and the ION parenchyma as the hypointense (dark) area within the hyperintense ring-shaped area. *A*, ION from a healthy individual; *B*, ION from a glaucoma patient.

All subjects were examined in supine position using a 3T MRI scanner (Magnetom TIM Trio; Siemens Co., Erlangen, Germany). This device allows head stabilization with foam cushions on both sides to minimize head motion. To ensure standardization and repeatability of the MRI scans, the axial plane of each MRI session was set parallel to the line from the anterior commissure to the posterior commissure on sagittal localizer images. For MRI scans of the ION, we combined two receiver coils: a 12-channel phased-array head coil and a single-loop surface coil placed anteriorly to the examined orbit and centered on the eye to boost MRI signal at the region of interest. ION screening included an ultrafast T2-weighted half-Fourier acquisition in single shot turbo spin-echo (HASTE) sequence oriented perpendicular to the ION in all planes, with the following parameters: repetition time (TR) = 1510 ms; echo time (TE) = 148 ms; number of excitations = 1; bandwidth = 195 Hz/pixel; field of view (FOV) = 126 x 180 mm^2^; matrix = 512 x 286; and slice thickness = 2 mm. In the T2-weighted images, cerebrospinal fluid was identified as a hyperintense ring (bright), and the ION parenchyma as a hypo-intense signal (dark) inside the hyperintense ring (Fig 1). To minimize eye movement and possible anatomical changes at the ION during image acquisition process, participants were required to fixate, in primary gaze, on a stationary target (black dot on white paper) positioned 100 cm horizontally-distant from the participant’s head, looking through a 45°-angled mirror placed on the head coil (to allow visualization of the target).

**Fig 2:**
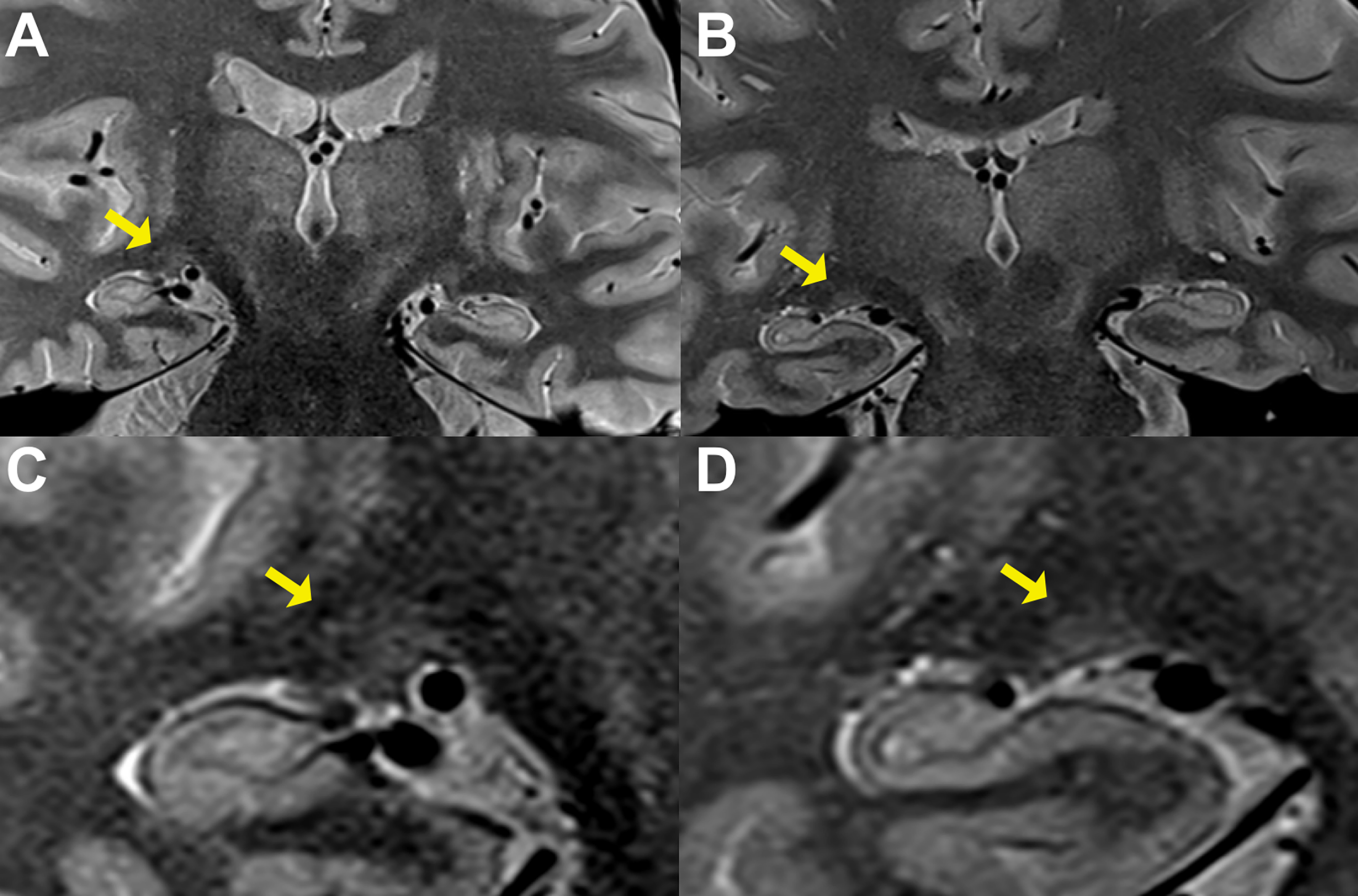
Magnetic resonance imaging scans showing the lateral geniculate nucleus (LGN). *A*, right LGN of a healthy individual (yellow arrow); *B*, right LGN of a glaucoma patient; *C*, zoomed-in image of the same LGN (yellow arrow) in image A; *D*, zoomed-in image of the same LGN (yellow arrow) in image B.

Additionally, LGN morphometry was evaluated using a 32-channel head coil of the same MRI device, in 2 separated sequences. First, a 3-dimensional T1-weighted magnetization-prepared rapid gradient-echo (3D-MPRAGE) sequence with: TR = 2340 ms; TE = 3.5 ms; inversion time = 1100 ms; number of excitations = 1; bandwidth = 190 Hz/pixel; FOV = 256 x 256 mm^2^, slice thickness = 1 mm; matrix = 512 x 512; and second, a coronal proton-density-weighted turbo spin-echo sequence with: TR = 2500 ms; TE = 19 ms; bandwidth = 205 Hz/pixel; FOV = 240 x 240 mm^2^, slice thickness = 2 mm; matrix = 314 x 448; number of excitations = 1 (Fig 2).

The boundaries of the ION, optic nerve sheath and LGN were manually delineated based upon consensus among three board-certified neuroradiologists (FE, MM and EAJ) masked from clinical data. Delineation and measurements were performed using OsiriX Imaging Software (freely available at http://www.osirix-viewer.com),[32] version 3.9.3, in a standardized magnification window of 700%. Neuroradiologists adopted a minimum image quality criteria to consider delineation of MRI image structures, as follows: 1) for the ION sheath and ION parenchyma, at least 75% of their borders should be promptly identified in the MRI image; and 2) for the LGN, 100% of its borders should be promptly identified in the MRI image. For this study, mean diameter and cross-sectional area of the ION segments at 5 mm, 10 mm, and 15 mm behind the eye, and base length, height and cross-sectional area of the LGN were the 3T MRI-derived continuous variables included in the analysis. Although LGN measurements had been performed in all 3T MRI scans in which LGN could be identified, we included in the statistical analyses only LGN parameters obtained from the scan that showed the largest LGN cross-sectional area. After delineation of the boundaries, LGN height was calculated as the distance perpendicular to the apex of the LGN convexity and the LGN base. Mean diameter of the ION was calculated as the average between the largest diameter and its perpendicular diameter to minimize possible bias caused by non-round shaped optic nerves.

### Statistical analysis

Descriptive statistics are presented with frequency tables or graphics, whereas estimates of center and dispersion are described, respectively, as mean and standard deviation (SD). BCVA was converted from Snellen scale to logarithm of the minimum angle of resolution (logMAR) for quantitative analysis. To control for dependencies between both eyes, both ION or both LGN of the same individual, generalized estimating equations (GEE)[33, 34] models were used assuming an interchangeable working correlation structure. We also used the bootstrap resampling method for clustered data using 1,000 replications,[35] adjusted for diagnosis (control or glaucoma), to estimate how well the data fit the statistical model, and calculating the coefficient of determination (R^2^).

Moreover, diagnostic ability of MRI parameters of the ION was assessed using receiver operating characteristic (ROC) curves to distinguish healthy eyes from eyes with glaucoma, taking into account the dependencies between eyes of the same individual. The area under the ROC curve (AUC) was used to summarize the diagnostic accuracy of each parameter.[36] For this analysis, SAP outcomes were used as the reference standard to discriminate healthy eyes from those with glaucoma. Of note, an AUC of 1.0 denotes a perfect discrimination between the two diagnostic possibilities (healthy or glaucoma), whereas an area of 0.5 represents chance discrimination. The method of DeLong et al. was applied to compare the best-performing AUC of devices used for structural evaluation.[37]

Computerized statistical analyses were performed using commercially available softwares (SPSS Statistics, version 20.0, IBM-SPSS, Chicago, IL; and Stata, version 13.0, Stata Corp., College Station, TX) and the alpha level was set at 5%.

## Results

In the glaucoma group, 15 (36.6%) patients had at least one eye with early disease, 17 (41.5%) patients had at least one eye with moderate disease, and 28 (68.3%) patients had at least one eye with advanced disease. There was no difference between fellow eyes in the glaucoma group regarding any functional or structural parameters (all, p≥0.071). The characteristics of the glaucoma group regarding disease severity in each eye are described in Table 2. Less than 15% of the glaucoma patients were assigned in the asymmetrical glaucoma subgroup, what indicated that most of glaucoma patients did not have remarkable functional damage asymmetry between eyes.

**Table 2:** Severities of visual field damage in both eyes of glaucoma subjects according to standard automated perimetry results and subgroup classification.

Better Eye	Poorer Eye	Subgroup	n (%)
Early / Moderate	Early / Moderate	Mid-Stage Glaucoma	13 (31.7)
Early	Severe	Asymmetric Glaucoma	6 (14.6)
Moderate / Severe	Severe	Advanced Glaucoma	22 (53.7)

Results from 3T MRI showed that the mean diameter and cross-sectional area of the ION were significantly different between glaucoma and control groups at 5 mm (both p≤0.011) and 10 mm (both p≤0.001) behind the globe, but not at 15 mm (both p≥0.067). Regarding the LGN, the only parameter that showed a significant difference between glaucoma and control groups was the LGN height (mean: 3.81±0.5 mm and 4.11±0.5 mm, respectively; p = 0.005) (Table 3), as illustrated in Fig 2. No significant differences were found with regard to base length or cross-sectional area of LGN between the two groups.

**Table 3:** Structural characteristics of the optic nerve sheath, intraorbital optic nerve and lateral geniculate nucleus measured with 3-Tesla magnetic resonance imaging based on the level of functional damage on standard automated perimetry.

Intracranial Structures	Glaucoma				Control	P values†						
	(n = 41)				(n = 12)							
	Overall	Early	Moderate	Advanced		P_1_	P_2_	P_3_	P_4_	P_5_	P_6_	P_7_
Optic nerve sheath												
- Mean diameter, mean (SD), mm												
• 5 mm*	5.42 (0.7)	5.37 (0.6)	5.45 (0.9)	5.25 (0.7)	5.47 (0.9)	0.39	0.558	0.374	0.309	0.496	0.736	0.238
• 10 mm*	4.52 (0.5)	4.54 (0.5)	4.59 (0.5)	4.28 (0.5)	4.82 (0.5)	0.034	0.354	0.720	0.052	0.366	0.112	0.010
• 15 mm*	3.79 (0.5)	3.86 (0.5)	4.07 (0.5)	3.62 (0.4)	4.13 (0.7)	0.132	0.317	0.163	0.183	0.756	0.004	0.043
- Cross-sectional area, mean (SD), mm^2^												
• 5 mm*	22.85 (7.1)	22.58 (7.4)	23.86 (9.1)	20.60 (5.3)	23.19 (7.9)	0.485	0.889	0.050	0.194	0.934	0.236	0.182
• 10 mm*	15.05 (3.9)	16.58 (7.2)	15.67 (3.7)	13.28 (3.5)	16.72 (3.4)	0.036	0.948	0.167	0.067	0.359	0.042	0.003
• 15 mm*	10.67 (2.7)	11.80 (2.8)	11.86 (2.8)	9.44 (2.1)	12.3 (4.3)	0.212	0.999	0.358	0.005	0.999	0.003	0.077
Intraorbital optic nerve												
- Mean diameter, mean (SD), mm												
• 5 mm*	3.08 (0.3)	3.40 (1.0)	3.09 (0.5)	2.97 (0.3)	3.29 (0.4)	0.011	0.636	0.176	0.048	0.119	0.323	0.002
• 10 mm*	2.68 (0.4)	2.77 (0.4)	2.97 (0.4)	2.47 (0.3)	3.1 (0.4)	<0.001	0.023	0.148	0.007	0.197	<0.001	<0.001
• 15 mm*	2.35 (0.4)	2.50 (0.3)	2.62 (0.3)	2.14 (0.3)	2.54 (0.7)	0.204	0.999	0.277	<0.001	0.999	<0.001	0.026
- Cross-sectional area, mean (SD), mm^2^												
• 5 mm*	7.34 (1.6)	7.49 (1.6)	7.41 (2.0)	6.91 (1.5)	8.51 (1.6)	0.003	0.074	0.893	0.116	0.047	0.310	0.001
• 10 mm*	5.97 (1.7)	8.59 (9.0)	7.28 (1.5)	4.94 (1.3)	7.57 (2.1)	0.001	0.657	0.487	<0.001	0.452	<0.001	<0.001
• 15 mm*	4.53 (1.4)	5.03 (1.0)	5.65 (1.4)	3.73 (0.9)	5.39 (1.9)	0.067	0.408	0.244	<0.001	0.919	<0.001	0.002
Lateral geniculate nucleus												
- Height, mean (SD), mm	3.81 (0.5)				4.11 (0.5)	0.005						
- Base length, mean (SD), mm	6.87 (0.8)				7.17 (0.8)	0.165						
- Cross-sectional area, mean (SD), mm^2^	16.02 (3.1)				17.07 (3.0)	0.209						

* Distance from the posterior sclera.

† Generalized estimating equations.

SD = Standard deviation.

1 = Difference between overall glaucoma and control groups.

2 = Difference between early glaucoma and control groups.

3 = Difference between early and moderate glaucoma subgroups.

4 = Difference between early and advanced glaucoma subgroups.

5 = Difference between moderate glaucoma and control groups.

6 = Difference between moderate and advanced glaucoma subgroups.

7 = Difference between advanced glaucoma and control groups.

When patients were divided into groups according to severity of functional damage on SAP, we observed differences dependent upon the distance from the globe. The ION mean diameter at 5 mm from the eyes with advanced glaucoma was statistically different from control eyes (p = 0.002) and from eyes with early glaucoma (p = 0.048). The ION cross-sectional area at 5 mm in the control group was different from eyes with moderate glaucoma (p = 0.047) and advanced glaucoma (p = 0.001). At 10 mm behind the globe, the ION dimensions in the advanced glaucoma group differed significantly from those of the other groups (all, p≤0.007), and the ION diameter from the control group different from those with early glaucoma (p = 0.023). Similarly to measurements at 10 mm, ION dimensions at the 15 mm in advanced glaucoma were statistically different from the other groups (p≤0.022).

Once height was the only LGN parameter showing statistical difference between glaucoma and control groups, we also investigated the relationship between this parameter and severity of SAP functional damage in ipsilateral and contralateral eyes. Considering ipsilateral damage, we observed significant difference between the control group and groups with moderate or advanced disease (p = 0.027 and p = 0.01, respectively). Based on the contralateral eye SAP results, the control group had significantly greater LGN height than the early and advanced glaucoma groups (p = 0.048 and p = 0.005, respectively).

In both control and glaucoma groups we observed a trend of decreased ION mean diameter and cross-sectional area as they were more posterior from the globe towards orbital apex. In the control group, there were statistically significant differences of ION dimensions between 5 mm and 10 mm from the globe, between 5 mm and 15 mm, and between 10 mm and 15 mm (p≤0.024), except for ION mean diameter between 5 mm and 10 mm from the eye (p = 0.096). In glaucoma group, each ION parameter was shown to be statistically different among the distinctive distances from the globe (all comparisons, p<0.001).

In the analyses of structure-structure and structure-function relationships, we observed that ION mean diameter and its cross-sectional area behaved similarly (Tables 4–7). In general, the ION parameters at 10 mm and 15 mm behind the eye correlated better with ocular structural parameters than those obtained closest to the eye. The ION parameters measured at 15 mm from the globe correlated positively with the neuroretinal rim area obtained on stereophotographs (0.1≤R^2^≤0.124; p≤0.004), but not with their CDR (Table 4).

**Table 4:** Relationship between 3-Tesla magnetic resonance imaging findings of the intraorbital optic nerve and optic disc stereophotograph outcomes.

Intraorbital Optic Nerve	Optic Disc Stereophotograph											
	Vertical CDR				Average CDR				Neuroretinal Rim Area			
	R^2^	P^†^	*β*	P^‡^	R^2^	P^†^	*β*	P^‡^	R^2^	P^†^	*β*	P^‡^
Mean diameter												
• 5 mm*	0.08	0.048	0.1	0.794	0.079	0.051	0.06	0.875	0.08	0.043	0.032	0.727
• 10 mm*	0.193	0.002	-0.234	0.533	0.192	0.002	-0.222	0.614	0.209	0.001	0.166	0.134
• 15 mm*	0.054	0.122	-0.569	0.153	0.053	0.138	-0.629	0.203	0.1	0.003	0.297	0.002
Cross-sectional area												
• 5 mm*	0.113	0.015	-0.533	0.724	0.113	0.012	-0.569	0.734	0.118	0.011	0.352	0.399
• 10 mm*	0.176	0.004	-2.219	0.199	0.175	0.006	-2.476	0.233	0.205	0.001	1.087	0.015
• 15 mm*	0.091	0.044	-2.559	0.067	0.092	0.043	-2.957	0.08	0.124	0.006	1.033	0.004

Bootstrap resampling method for clustered data, 1,000 replications, adjusted for diagnosis

* Distance from the eye.

† Significance level of the statistical model, adjusted for diagnosis.

‡ Significance level of the participation of the independent variable in the statistical model

R^2^ = coefficient of determination.

*β* = coefficient of the linear regression model.

CDR = cup-to-disc ratio.

**Table 5:** Relationship between 3-Tesla magnetic resonance imaging findings of the intraorbital optic nerve and spectral-domain optical coherence tomography outcomes.

Intraorbital Optic Nerve	Spectral-Domain Optical Coherence Tomography																	
	Vertical CDR				Average CDR					Neuroretinal Rim Area				RNFLT				
	R^2^	P^†^	*β*	P^‡^	R^2^	P^†^		*β*	P^‡^	R^2^	P^†^	*β*	P^‡^	R^2^	P^†^		*β*	P^‡^
Mean diameter																		
• 5 mm*	0.084	0.021	-0.294	0.574	0.087	0.028		-0.406	0.416	0.114	<0.001	0.31	0.023	0.087	0.025		0.003	0.274
• 10 mm*	0.199	0.001	-0.517	0.295	0.198	0.003		-0.495	0.31	0.264	<0.001	0.555	0.001	0.284	<0.001		0.014	<0.001
• 15 mm*	0.065	0.069	-0.968	0.119	0.061	0.175		-0.92	0.209	0.16	<0.001	0.704	<0.001	0.153	0.002		0.015	0.001
Cross-sectional area																		
• 5 mm*	0.121	0.003	-2.023	0.396	0.125	0.002		-2.462	0.284	0.158	<0.001	1.722	0.003	0.128	0.003		0.023	0.184
• 10 mm*	0.176	0.006	-3.071	0.163	0.17	0.019		-2.401	0.373	0.249	<0.001	2.61	<0.001	0.319	<0.001		0.077	<0.001
• 15 mm*	0.1	0.058	-3.962	0.055	0.101	0.111		-4.024	0.091	0.226	<0.001	2.808	<0.001	0.266	<0.001		0.07	<0.001

Bootstrap resampling method for clustered data, 1,000 replications, adjusted for diagnosis

* Distance from the eye.

† Significance level of the statistical model, adjusted for diagnosis.

‡ Significance level of the participation of the independent variable in the statistical model

R^2^ = coefficient of determination.

*β* = coefficient of the linear regression model.

CDR = cup-to-disc ratio.

RNFLT = peripapillary retinal nerve fiber layer thickness.

**Table 6:** Structure-function relationship between 3-Tesla magnetic resonance imaging findings of the intraorbital optic nerve and standard automated perimetry outcomes.

Intraorbital Optic Nerve	Standard Automated Perimetry											
	MD				VFI				Linear MS			
	R^2^	P^†^	*β*	P^‡^	R^2^	P^†^	*β*	P^‡^	R^2^	P^†^	*β*	P^‡^
Mean diameter												
• 5 mm*	0.144	<0.001	0.011	0.001	0.143	<0.001	0.003	0.002	0.136	<0.001	<-0.001	0.005
• 10 mm*	0.325	<0.001	0.019	<0.001	0.312	<0.001	0.006	<0.001	0.3	<0.001	<-0.001	<0.001
• 15 mm*	0.232	<0.001	0.022	<0.001	0.228	<0.001	0.007	<0.001	0.202	<0.001	<-0.001	<0.001
Cross-sectional area												
• 5 mm*	0.174	<0.001	0.05	0.001	0.172	<0.001	0.015	<0.001	0.164	<0.001	<-0.001	0.003
• 10 mm*	0.329	<0.001	0.09	<0.001	0.31	<0.001	0.026	<0.001	0.283	<0.001	<-0.001	<0.001
• 15 mm*	0.289	<0.001	0.083	<0.001	0.288	<0.001	0.025	<0.001	0.268	<0.001	<-0.001	<0.001

Bootstrap resampling method for clustered data, 1,000 replications, adjusted for diagnosis

* Distance from the eye.

† Significance level of the statistical model, adjusted for diagnosis

‡ Significance level of the participation of the independent variable in the statistical model

R^2^ = coefficient of determination.

*β* = coefficient of the linear regression model.

MD = mean deviation.

VFI = visual field index.

MS = mean sensitivity.

**Table 7:** Structure-function relationship between 3-Tesla magnetic resonance imaging findings of the intraorbital optic nerve and frequency doubling technology outcomes.

Intraorbital Optic Nerve	Frequency Doubling Technology							
	MD				Linear MS			
	R^2^	P^†^	*β*	P^‡^	R^2^	P^†^	*β*	P^‡^
Mean diameter								
• 5 mm*	0.182	<0.001	0.017	<0.001	0.162	<0.001	<-0.001	<0.001
• 10 mm*	0.332	<0.001	0.025	<0.001	0.319	<0.001	<-0.001	<0.001
• 15 mm*	0.196	<0.001	0.026	<0.001	0.188	<0.001	<-0.001	<0.001
Cross-sectional area								
• 5 mm*	0.211	<0.001	0.08	<0.001	0.179	<0.001	<-0.001	0.004
• 10 mm*	0.347	<0.001	0.121	<0.001	0.304	<0.001	<-0.001	<0.001
• 15 mm*	0.258	<0.001	0.098	<0.001	0.26	<0.001	<-0.001	<0.001

Bootstrap resampling method for clustered data, 1,000 replications, adjusted for diagnosis

* Distance from the eye.

† Significance level of the statistical model, adjusted for diagnosis.

‡ Significance level of the participation of the independent variable in the statistical model.

R^2^ = coefficient of determination.

*β* = coefficient of the linear regression model.

MD = mean deviation.

MS = mean sensitivity.

The OCT ONH rim area was the only ocular structural parameter that correlated positively with all ION measurements at any of the distances behind the eye, especially at 10 mm from the eye (0.249≤R^2^≤0.264, p≤0.001; Table 5). Also, the OCT average peripapillary RNFLT correlated positively with the ION parameters at 10 mm and 15 mm, reaching the strongest level of correlation with the ION cross-sectional area at 10 mm (R^2^ = 0.319, p<0.001; Table 5). There was no association between ION parameters and CDR measured with OCT. Surprisingly, no association between ION parameters with CSLO parameters reached statistical relevance (all, p≥0.069).

Interestingly, the ION mean diameter and its cross-sectional area at 5 mm, 10 mm, and 15 mm behind the globe correlated significantly with all SAP and FDT parameters (all, p≤0.004), reaching the strongest level of association at 10 mm (0.283≤R^2^≤0.347, p<0.001; Tables 6 and 7).

LGN parameters were not significantly associated with any of the studied ipsilateral and contralateral structural parameters (OCT, CSLO or stereophotograph variables), nor with any ipsilateral or contralateral functional (SAP or FDT) parameters. Moreover, there was no significant association between LGN parameters and the estimated functional data that took into account the appropriate afferents at the LGN.

**Fig 3:**
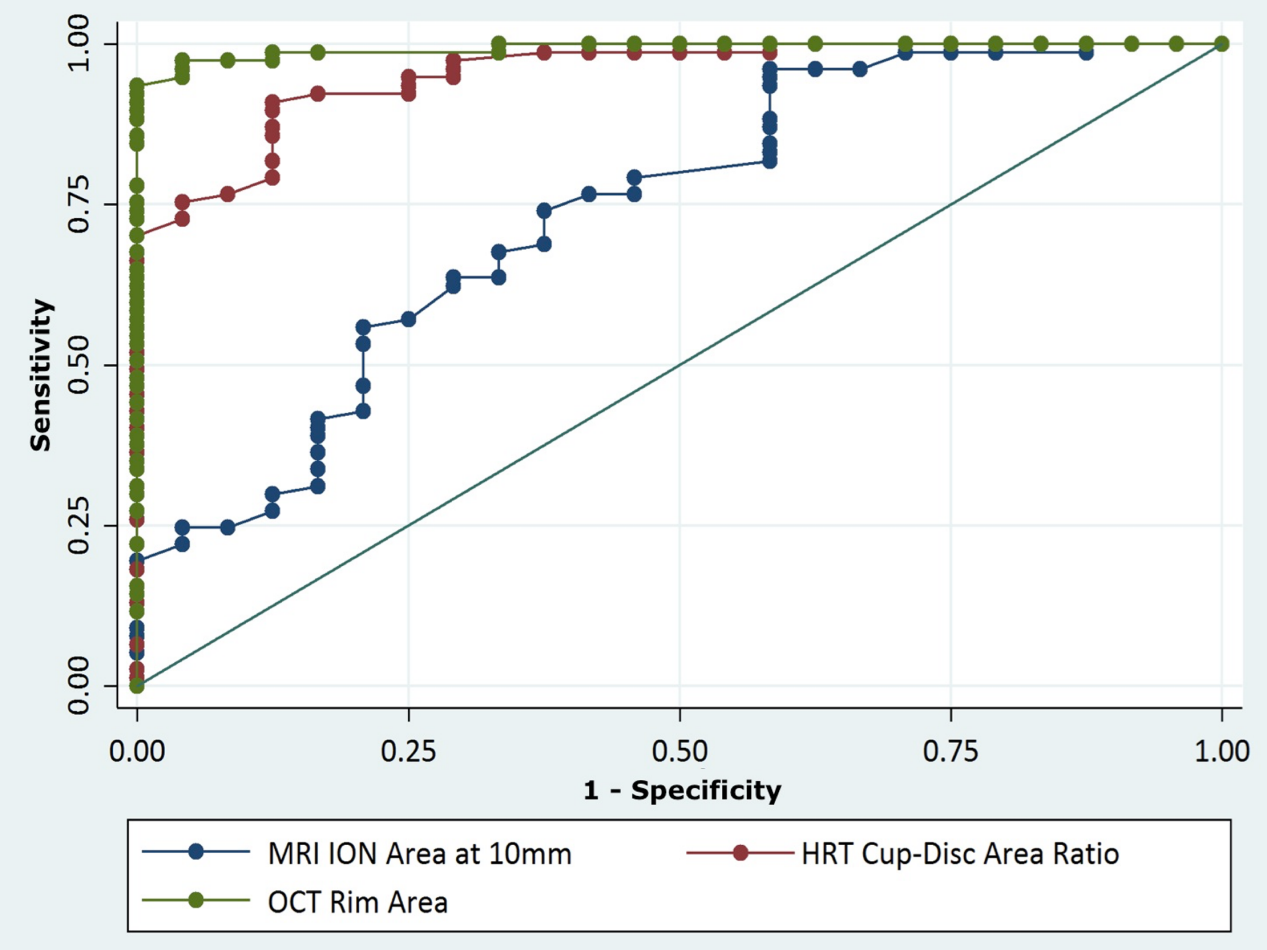
Receiver operating characteristic curves for the best-performing parameters of the different devices used in the study to discriminate glaucomatous from healthy eyes. MRI = magnetic resonance imaging; ION = intraorbital optic nerve; HRT = confocal scanning laser ophthalmoscopy;OCT = spectral-domain optical coherence tomography.

In general, 3T-MRI ION parameters were not accurate in discriminating healthy from glaucomatous eyes, particularly if compared to conventional structural parameters, such as OCT or CSLO. The ION mean diameter at 10 mm and the ION cross-sectional areas at 5 mm and 10 mm had significant, but relatively poor ability to differentiate healthy from glaucomatous eyes {AUC = 0.639 [95% confidence interval (CI), 0.507–0.771]; AUC = 0.720 (95% CI, 0.599–0.841) and AUC = 0.738 (95% CI, 0.618–0.858), respectively}. Of note, the parameters from the conventional structural tests for glaucoma that presented the best-performing AUC were OCT-derived rim area and CSLO-derived cup-to-disc area ratio [AUC = 0.993 (95% CI, 0.983–1.0) and AUC = 0.955 (95% CI, 0.917–0.993), respectively]. In a comparison between the best-performing AUC of each device (OCT, CSLO and 3T MRI), we observed that ION cross-sectional area at 10 mm had significant poorer ability than OCT-derived rim area and CSLO-derived cup-to-disc area ratio to differentiate healthy from glaucomatous eyes (p<0.001 and p = 0.002, respectively; Fig 3).

## Discussion

In this study we analyzed the structural aspects of 3T MRI-measured ION and LGN and their association with the level of glaucomatous damage based on conventional functional or structural tests. First, we showed that not only ION parameters but also LGN height are reduced in glaucoma, which is consistent with previous studies.[3, 4, 7–11, 15, 17–19] In addition, we observed a positive correlation of OCT rim area and RNLFT with ION mean diameter and its cross-sectional area at different distances from the eye, mainly the most posterior ones. Furthermore, we demonstrated a positive correlation between ION measurements and functional parameters obtained from SAP and FDT. Nevertheless, LGN measurements were not associated with any tested functional or structural parameters in glaucoma.

Histological studies using human specimens demonstrated that the ION diameter was reduced in glaucoma and correlated with the number of retinal ganglion cell axons.[3, 4] Reduced ION diameter in glaucoma was also observed in other *in vivo* studies using different imaging technologies.[15, 17, 18, 38–41] For instance, in an ecographic study with 49 glaucomatous eyes and 90 control ones, Beatty et al. found smaller ION diameter and cross-sectional area in glaucoma patients when compared to normal participants, and suggested that ION cross-sectional areas inferior to the 95% CI of normality should be considered pathological.[38] However, given the limited spatial resolution of the imaging devices used in those studies, differentiation between ION and optic nerve sheath may have been challenging and could have led to inaccuracy of measurements.

New 3T MRI systems are known by their imaging advancements, such as increased image acquisition speed, better spatial and contrast image resolution, and therefore, provide improved image quality.[42] The result is an enhanced detection of boundaries between the optic nerve sheath, the cerebrospinal fluid space, and the actual ION parenchyma.[14, 43] Using this method, we found smaller ION dimensions at different distances from the eye in glaucomatous relative to normal ones, which is consistent with previous MRI studies.[15, 21, 44] In one such MRI study, Chen et al. described a smaller cross-sectional area of the ION in patients with advanced glaucoma compared to an age- and sex-matched control group.[44] Using a different MRI protocol, Ramli et al. observed a significantly lower ION volume in advanced glaucoma compared to mild glaucoma or control groups, but no difference was found between mild glaucoma and control groups.[21] Our results are, at least in part, consistent with those studies, since the advanced glaucoma group had smaller ION diameter and cross-sectional area than the control group in all studied distances from the eye. This difference was also observed when the advanced glaucoma group was compared with the mild glaucoma group, except for the ION cross-sectional area at 5 mm from the eye.

In addition, we observed in both glaucoma and control groups a decrease of the ION diameter and its cross-sectional area as a function of distance from the eye, which is consistent to histological and MRI findings.[15, 18, 20, 43] In a study with 23 normal individuals and 3 human specimens, Karim et al. demonstrated a decrease in the ION diameter along its course towards the orbital apex using both histological sections and MRI.[20] They also showed a close quantitative agreement between measurements from those techniques, which suggests a high accuracy of MRI measurements.[20] In addition, the authors observed a progressive and significant decrease in the amount of ION connective tissue from the anterior to posterior region of the orbit, but the proportion of neuronal tissue in the ION was independent of the distance from the globe. They postulated that the ION thinning along its course was related to a reduction of the amount of connective tissue.[20] When evaluating glaucoma patients, MRI studies also described that ION diameters were smaller near the orbital apex when compared to cross-sectional images closer to the eye.[15, 18] Assuming the ION is thicker near the globe due to a larger amount of connective tissue, it is possible that this structural arrangement may be a protective mechanism for the ION fibers undergoing mechanical stress during ocular movements.

In our study, the distal location of the ION was significantly correlated with the rim area of the ONH obtained with different ocular structural tests. Of note, the neuroretinal rim is one of the most valuable features of the ONH in glaucoma evaluation, and estimation of the rim area is an indirect measure of the amount of neural tissue in the ONH.[3, 45–47] Balazsi et al. showed that the neuroretinal rim measured with stereophotographs was the only parameter capable of differentiating normal individuals, glaucoma suspects, and mild glaucoma.[45] Although just a few *in vivo* studies focused on the relationship between ONH rim area and ION parameters measured with devices available in daily practice,[39, 48, 49] our results are consistent with their findings. Using echography, Dichtl and Jonas showed that the ION thickness was associated with the neuroretinal rim area assessed using optic disc photographs,[39] while Beatty et al. reported a significant correlation between ION measurements and CLSO rim area in glaucomatous or ocular hypertensive patients.[48] Nevertheless, echography measurements of the ION are often obtained at its proximal location,[38–40] and may vary considerably when compared to MRI findings.[43] In a study using diffusion tensor MRI, Chang et al. observed a significant association between presumed ION fiber integrity changes and the ONH rim area assessed by CSLO.[49] In our 3T MRI study, we found that the ION dimensions at its distal location could be used to estimate the ONH rim area measured with OCT or stereophotographs. Furthermore, we observed a positive correlation between the ION distal segments (10 mm and 15 mm from the eye) and the peripapillary RNFLT measured with OCT, which is at least in part consistent with previous reports.[15, 18] Using 1.5 Tesla MRI in normal-tension glaucoma patients, Zhang et al. described that the strongest correlation occurred between the ION diameter at 15 mm behind the eye and the OCT peripapillary RNFLT.[18] In a 3T MRI study, Lagrèze et al. found a positive correlation between the ION mean diameter at 15 mm behind the globe and RNFLT measured with scanning laser polarimetry.[15] Ramli et al. reported a moderate correlation between OCT-derived RNFLT and the ION volume,[21] the estimation of which was also influenced by the location where the ION volume was measured. The histological arrangement of the ION may help explain the stronger structural associations found in the most distal segments. As described by Karim et al., there is less connective tissue within the distal sections of the ION when compared to more proximal segments.[20] This may suggest that the ION measurements at more distal locations may be more representative of the actual amount of neuronal tissue within the ION. In addition, one could infer that the distal ION segments may be more stable and less susceptible to movement artifacts during MRI image acquisition, as it is closer to the optic canal of the sphenoid bone. This could lead to more accurate measurements of the ION and, consequently, to stronger structural correlations in its distal locations relative to the more proximal ones.

Previous studies suggested an association between ION structural properties and visual field status in glaucoma, which is consistent with our results.[17, 18, 50] Kashiwagi et al. observed not only a smaller ION diameter in glaucoma, but also a significant association between SAP MD and the ION diameter using a 1.5 Tesla MRI.[17] Using similar MRI system, Zhang et al. observed higher correlation coefficients between ION and mean perimetric loss in the orbital apex than near the globe.[18] Lagrèze et al. observed a stronger structure-function correlation for ION measurements 15 mm behind the eye using 3T MRI.[15] In another 3T MRI study, Omodaka et al. suggested that the average of the ION cross-sectional areas measured at 3 distinct locations correlated significantly with SAP MD.[50] We detected that not only the ION distal location, but any location of the ION correlated significantly with current methods for functional evaluation in glaucoma, namely SAP and FDT. To the best of our knowledge, an association between ION glaucomatous damage and functional deterioration evaluated by FDT has not yet been reported in the literature. Of note, FDT perimetry had higher sensitivity and specificity than other screening tests to detect glaucoma, even among eyes without functional damage on SAP (preperimetric glaucoma),[51, 52] and outperformed SAP in detecting the onset of functional glaucomatous damage in the early stage of the disease.[53] Since we did not include eyes with preperimetric glaucoma, future MRI studies ought to test if FDT could be employed in investigations of the ION in this group of patients. Moreover, in the study by Lamparter et al.,[54] FDT correlated better with ONH parameters measured by CSLO than SAP. In our study, the strongest level of structure-function association was seen between the ION cross-sectional area at 10 mm behind the eye and the FDT global index, although SAP and FDT had similar performances in their correlation with the ION properties. SAP and FDT also performed similarly when correlated with OCT-derived RNFLT in the study by Pinto et al.,[55] what supports our findings, as fibers within RNFL converge at the ONH to comprise the ION.[56]

Regarding LGN damage, transsynaptic degeneration was postulated in glaucomatous optic neuropathy once metabolic changes,[57] dendritic alteration,[58] and neuronal loss were observed within magnocellular and parvocellular LGN layers in experimental glaucoma, leading to shrinkage of the LGN.[10, 11, 59] Gupta et al. showed that the LGN height measured with MRI in normal subjects was comparable to histomorphometric measurements of the LGN obtained from normal post-mortem specimens,[7] which suggests a high accuracy and reliability of this technology for LGN assessment. In different studies, LGN height was significantly decreased in glaucoma patients compared to healthy individuals.[16, 19, 22] Our findings were consistent with those studies, as we found that LGN height was the only LGN parameter reaching statistical significance to differentiate between glaucoma subjects and age- and sex-matched healthy controls. Hence, LGN height may be a key parameter for the assessment of glaucomatous damage to the CNS.

Given the particular anatomical arrangement of the anterior visual pathway, retinal ganglion cells axons from one eye partially decussate at the optic chiasm following a particular pattern,[60, 61] and synapse at the ipsilateral or contralateral LGN depending on their retinal topography.[56] More specifically, the ganglion cell axons from the temporal retina of the ipsilateral eye synapse in layers 2, 3, and 5, while axons from the nasal retina of the contralateral eye synapse in layers 1, 4, and 6. Based on cell morphology within each LGN layer, layers 1 and 2 are classified as magnocellular, whereas layers 3–6, as parvocellular.[56] On the other hand, functional or ocular structural data are often summarized into numerical indices, which may in turn not represent the entire complexity of the structural and functional arrangements that exist as fibers travel from the retina to the LGN. Thus, a significant relationship between LGN measurements and any functional or ocular structural parameter may not be easily revealed. This may explain in part the inconsistency among the few studies that addressed this issue,[16, 22, 62] including ours. In a 3T MRI study, Dai et al. suggested a possible correlation between glaucoma severity based on SAP results and LGN measurements, such as volume and height.[16] In another study, LGN height was associated with ipsilateral and contralateral CDR assessed with fundus photography and OCT-derived peripapillary RNFLT.[22] Using 7 Tesla MRI, Lee et al. found a positive correlation only between computer-estimated LGN volume and the combined thickness of two inner retinal layers obtained in the macular region of the contralateral eye, while no association was found using peripapillary RNFLT of either eye.[62] Although our patients were considerably older than those in the aforementioned studies, we did not find any significant association between LGN parameters and ocular structural or functional variables, even when appropriate afferents were considered. One should note there are two main types of neurons within the LGN: interneurons, which are confined to the LGN, and relay neurons, which synapse to the visual cortex. Interneurons were observed in a larger proportion at the magnocellular than in parvocellular layers,[63] and were suggested to be relatively more resistant to transneuronal degeneration as they expressed less atrophic changes compared to relay neurons after enucleation.[64] Further, in experimental glaucoma, relay neurons in parvocellular layers of the LGN underwent significantly more shrinkage than relay neurons in magnocellular layers, as described by Yücel et al.[13] This may help explain the absence of correlation between FDT and LGN parameters, once FDT emphasizes the response characteristics of the magnocellular visual pathway,[25] and also the reason SAP and FDT had similar performances when their correlations with LGN parameters were investigated. In addition, LGN height is mostly dependent of the hilum (central region), which receives macular retinal ganglion cells inputs in all those six layers. It is important to address that the macula has a high concentration of midget ganglion cells, which are linked to the parvocellular visual pathway.[12] Although the macula is a relatively small retinal area, its representation in the LGN is quite extensive, constituted by an area occupying two thirds or more of the LGN central portion.[65] In our study, we investigated structure-structure correlations between MRI results and ONH properties only, thus excluding the macula. Nevertheless, our results do not necessarily indicate that glaucomatous damage at the LGN is independent of ocular alterations. One possible explanation to our findings is that glaucomatous damage to ocular structures may not occur at the same time or could even precede abnormalities observed within the LGN and visual cortex as well. The same rationale could be applied to the interpretation of the lack of association between LGN parameters and visual function, as visual field defects in glaucoma are topographically correlated with ocular structural changes, including RNFL loss and ONH damage.[1, 2, 66] Eventually, time of disease might be an important factor, thus such temporal relationships warrant future investigation. The knowledge regarding transsynaptic degeneration in glaucoma comes mostly from experimental models, in which glaucomatous changes are often induced by fast IOP elevation after trabecular meshwork destruction in non-human eyes.[8, 57, 67] Consequently, a rapid neural tissue damage occurs and may not accurately depict the chronic neuronal changes seen in living POAG patients, which can often take a long time (months or years) to manifest detectable structural or functional changes. A longitudinal assessment of glaucomatous patients using MRI may provide relevant information about glaucoma-related transsynaptic degeneration in the living human optic pathway.

Notably, it is important to stress that the aim of our study was not to evaluate 3T MRI as a tool for glaucoma diagnosis, but rather to analyze possible correlations between conventional glaucoma evaluation tools (structural and functional testing) and intracranial alterations, in order to ascertain whether ocular damage in glaucoma could predict the loss of ION and/or LGN tissue. Our study has some limitations. First, imaging of the intracranial anatomy using structural MRI is particularly challenging, since its resolution *in vivo* is limited by physiological noise, including vessel pulsation, respiration and head movement.[68] IONs are small and mobile structures and may have a non-linear configuration, thus it is possible that some movement artifacts may have occurred. However, we minimized them by using a fixation target in primary gaze and by obtaining ION scans perpendicularly to its axis, to reduce the influence of non-perpendicular cross-sectional ION images, which could have led to measurement inaccuracy. In addition, MRI is usually limited in resolving structures on the millimeter scale.[68] Although the LGN margins were completely identified in our study, the 6 neuronal layers of the LGN, whose sizes are less than 1 mm thick each, were not reliably distinguished neither in our study nor in previous neuroimaging studies.[16, 19, 22] Second, it is possible that our MRI methodology may have enhanced the image quality of the ION at 10 mm from the eye, since combined signals from a surface loop coil and a 12-channel head coil were used to acquire images for ION measures. This could explain the stronger correlations observed at this ION location. Third, the study sample was relatively small, and may have been underpowered to determine associations between ION or LGN parameters and functional or ocular structural testing. Nevertheless, in this age- and sex-matched comparison, our sample was larger than some previous MRI studies.[15, 22, 44] Finally, the majority of our coefficients of determination showed weak to moderate correlations. One should perceive that the association models we used in our study took into consideration just one independent variable adjusted for the diagnosis. Thus, it is possible that structure-structure or structure-function correlations in glaucoma involving peripheral and CNS anatomies may be influenced by other factors not included in our models and which remain unknown.

In summary, ION dimensions presented, in general, mild to moderate correlation with functional and ocular structural parameters. Although functional parameters correlated with the ION dimensions at any distance from the eye, we detected moderate associations of SAP or FDT with the ION dimensions at 10 mm and 15 mm from the eye. Ocular structural parameters also correlated better with the ION dimensions closer to the orbital apex, especially the OCT-derived neuroretinal rim area and RNFLT. However, even though we confirmed that glaucoma patients had decreased LGN height compared to age- and sex-matched healthy subjects, 3T MRI-measured LGN parameters were not correlated with functional or ocular structural parameters. Possibly, correlation analyses in glaucoma that include peripheral and CNS structures may be influenced by other factors that still remain unclear. On the other hand, 3T MRI may help monitoring glaucomatous damage in the peripheral and CNS. Future longitudinal MRI studies are warranted to investigate whether temporal relationships could enhance structure-function and structure-structure associations linked to the LGN.

## Supporting information

S1 File Data set. (XLSX)
